# Interaction of Cu(II) and Ni(II) with Ypk9 Protein Fragment *via* NMR Studies

**DOI:** 10.1155/2014/656201

**Published:** 2014-03-24

**Authors:** Massimiliano Francesco Peana, Serenella Medici, Alessia Ledda, Valeria Marina Nurchi, Maria Antonietta Zoroddu

**Affiliations:** ^1^Department of Chemistry and Pharmacy, University of Sassari, Via Vienna 2, 07100 Sassari, Italy; ^2^Department of Chemical and Geological Sciences, University of Cagliari, Cittadella Universitaria, 09042 Monserrato, Italy

## Abstract

P_1_D_2_E_3_K_4_H_5_E_6_L_7_ (PK9-H), a fragment of Ypk9, the yeast homologue of the human Park9 protein, was studied for its coordination abilities towards Ni(II) and Cu(II) ions through mono- and bi-dimensional NMR techniques. Both proteins are involved in the transportation of metal ions, including manganese and nickel, from the cytosol to the lysosomal lumen. Ypk9 showed manganese detoxification role, preventing a Mn-induced Parkinsonism (PD) besides mutations in Park9, linked to a juvenile form of the disease. Here, we tested PK9-H with Cu(II) and Ni(II) ions, the former because it is an essential element ubiquitous in the human body, so its trafficking should be strictly regulated and one cannot exclude that Ypk9 may play a role in it, and the latter because, besides being a toxic element for many organisms and involved in different pathologies and inflammation states, it seems that the protein confers protection against it. NMR experiments showed that both cations can bind PK9-H in an effective way, leading to complexes whose coordination mode depends on the pH of the solution. NMR data have been used to build a model for the structure of the major Cu(II) and Ni(II) complexes. Structural changes in the conformation of the peptide with organized side chain orientation promoted by nickel coordination were detected.

## 1. Introduction

In a recent study Ypk9, the yeast orthologue to Park9 protein (alias ATP13A2), a member of the P_5_-type ATPase family, showed to be involved in yeast protection against the effects of potentially toxic divalent cations [1]. Deletion of this gene led to growth defects when cells were exposed to different metal ions, but the effect was even more pronounced for Mn(II), in whose presence cells did not grow at all. In this way the role of the protein was connected to protection against noxious metal ions, and in its human form, when mutated, to Mn-induced Parkinsonism [2, 3]. Deletion of Ypk9 in yeast cells caused growth defects also in the presence of different other metal ions, including Ni(II). The whole protein is rich in coordinating residues, and the P_5_-type ATPase family has been rubricated as a “cation transporter”, although its role has not yet been fully clarified. It can thus be possible that mutations on Ypk9 and/or Park9 may have a role in cell sensitivity towards different cations. So, the effectiveness of metal binding to proper sequences within the proteins should be confirmed.

**Figure 1:**
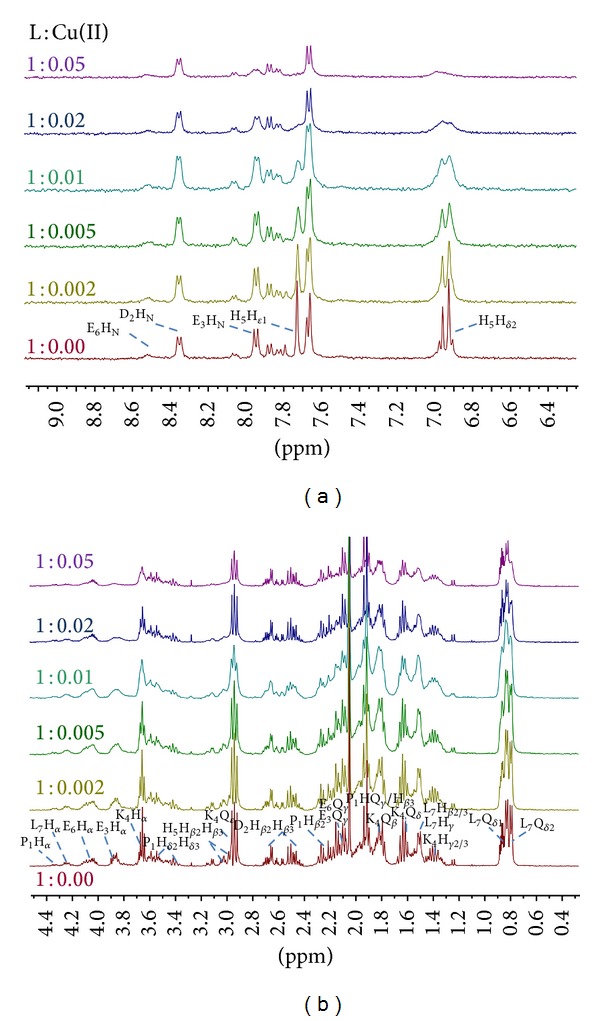
Stacked image of ^1^H NMR spectra for the aromatic (a) and aliphatic (b) regions for the PK9-H peptide, 2.5 mM, and pH 7.6, *T* 298 K, with increasing amounts of Cu(II) from 1 : 0.00 to 1 : 0.05 metal to ligand molar ratios.

For this study, we chose a fragment, P_1_D_2_E_3_K_4_H_5_E_6_L_7_ (PK9-H from now on, Figure  1s), containing a histidine residue whose role is to act as an anchoring site for metal ions. The same fragment had already been tested for Mn(II), Cu(II), and Zn(II) binding, giving interesting results [4, 5] and showing an effective interaction with these metals.

In the present paper, we complete the previous study on Cu(II) coordination, extend the scope of our research also to Ni(II) ions, and discuss the obtained results. Cu(II) was chosen for this investigation because it is involved in many biological processes within our organism, so its trafficking is of vital importance for a good functioning of the body [6–8]. Since Ypk9, as a P_5_-type ATPase, may be involved in divalent cations transportation, we cannot exclude that copper may be one of the metals carried by this protein. On the other hand, Ni(II) was also tested because it is implied in a number of pathologies, including allergies and cancer [9–11], and causes noxious effects on different other processes [12, 13], but as a divalent cation, it may be too transported by the same ATPase, noting that recently Park9 protein was shown to exert protection properties against nickel toxicity [14, 15].

From a chemical point of view, Ni(II) could work as a diamagnetic probe at a relatively high pH value (pH above 8) [16], and for this reason, it is utterly valuable in the study* via* NMR techniques of metal binding sites, where Cu(II), being paramagnetic, cannot always give enough information.

## 2. Experimental

### 2.1. Peptide Synthesis

The peptide PDEKHEL was chemically synthesized using solid-phase Fmoc (fluoren-9-ylmethoxycarbonyl) chemistry in an Applied Biosystems Synthesizer [17]. PDEKHEL was N-terminally acetylated and C-terminally amidated in order to mimic this region of Park9-Ypk9 within the full-length protein. The peptide was removed from the resin and deprotected before purification by reverse-phase HPLC. Fractions were collected and analyzed by MALDI-TOF MS. Fractions containing the peptide of the expected molecular weight were then pooled and lyophilized.

### 2.2. NMR Measurements

NMR experiments were performed on a Bruker Ascend 400 MHz spectrometer equipped with a 5 mm automated tuning and matching broad band probe (BBFO) with *z*-gradients.

Samples used for NMR experiments were in the range 2–2.5 mM in concentration and dissolved in 90/10 (v/v) H_2_O/D_2_O. All NMR experiments were performed at 298 K in 5 mm NMR tubes. 2D ^1^H-^13^C heteronuclear correlation spectra (HSQC) were acquired using a phase-sensitive sequence employing Echo-Antiecho-TPPI gradient selection with a heteronuclear coupling constant *J*_XH_ = 145 Hz and shaped pulses for all 180° pulses on f2 channel with decoupling during acquisition; sensitivity improvement and gradients in back-inept were also used [18–20].

Relaxation delays of 2 s and 90° pulses of about 10 *μ*s were applied for all experiments. Solvent suppression for 1D and TOCSY experiments was achieved using excitation sculpting with gradients. The spin-lock mixing time of the TOCSY experiment was obtained with MLEV17 [21].

^1^H-^1^H TOCSYs were performed using mixing times of 60 ms. ^1^H-^1^H ROESY spectra were acquired with spin-lock pulses duration in the range 200–250 ms [22].

The assignments of the peptide resonances were made by a combination of mono- and bidimensional and multinuclear NMR techniques ^1^H-^1^H TOCSY, ^1^H-^13^C HSQC, and ^1^H-^1^H ROESY at different pH values.

All NMR data were processed with TopSpin (Bruker Instruments) software and analyzed by Sparky 3.11 [23] and MestRe Nova 6.0.2 (Mestrelab Research S.L.) programs.

### 2.3. Structural Calculations

Structure calculations for the peptide-copper complex were performed on the basis of the experimental evidences obtained from UV-Vis and NMR results. Cu(II) contact map and metal-to-donor atoms distance obtained from analogous systems [24, 25] were used to model the Cu(II) complexes in a distorted tetragonal geometry, with an in plane 4N {N_*Im*⁡_, 3N^−^} chromophore, according to our spectroscopic results (this work and [5]).

Structure calculations for the peptide-nickel complex were performed on the basis of the experimental evidences plus ROE cross-correlations observed in 2D ^1^H-^1^H ROESY spectra. Due to the high pH value needed for the 4N1O {N_*Im*⁡_, 3N^−^, O^−^} complex formation (pH 10.5), no signals were detected in the aromatic region for the labile amide H^N^ aromatic protons, except for the imidazole protons H*ε*_1_ and H*δ*_2_ of the histidine ring. A structure determination has been carried out for the residues directly involved in the complex formation and in its close proximity (D_2_E_3_K_4_H_5_E_6_) for which significant ROEs were clearly detected. ROESY results were used as input data for structure calculations. The 2D cross-peaks of ^1^H-^1^H ROESY spectra of the peptide-Ni(II) system at 1 : 0.9 molar ratio were assigned and the intensities transformed into the maximal distances using the following method. Upper bounds *u* on the distance between two correlated hydrogen atoms were derived from the corresponding ROESY cross peak volumes *V* according to calibration curves *V* = *k*/*u* ^6^, with a constant *k* determined by using the cross-peak intensity H*δ*_2_-H*ε*_1_ of histidine imidazole aromatic protons as reference (*u* = 4.25 Å) [26]. The geometric constraints were set up, in agreement with the spectroscopic evidences, for a pentacoordinated {N_*Im*⁡_, 3N^−^, O^−^} species in a square pyramidal arrangement (*spy*) by fixing the Ni(II) ion with the four nitrogen donor atoms (N_*Im*⁡_, 3N^−^) in equatorial position and with the axial position occupied by the carboxylated oxygen (O^−^) of Asp-2. Ni(II) binding to the nitrogen of the Glu-3(N^−^)-Lys-4(N^−^)-His-5(N_*Im*⁡_, N^−^) sequence (square base of the pyramid) was restrained to the geometry encountered in the X-ray structure of the Ni(II)(Glycyl-Glycyl-Alpha-Hydroxy-D,L-Histamine)*·*3H_2_O [27, 28] with a calculation method already shown elsewhere [29].

Molecular mechanics geometry optimizations were obtained by an AMBER force field implemented in HyperChem(tm) 8.0.7 molecular modeling software. For energy minimization, the Polak Ribiere (conjugate gradient) algorithm was used to find the minimum within the HyperChem package. Models of the most likely coordination sphere for Cu(II) and Ni(II) species were generated with Chimera (YASARA Biosciences).

## 3. Results and Discussion

### 3.1. Cu(II) Complexes

The interaction of PK9-H fragment with Cu(II) ions had been partially studied in a previous paper [5], by using potentiometric methods and spectroscopic techniques such as NMR and UV-Vis. From potentiometric measurements, the species distribution diagram, depending on the pH of the solution, had been obtained; it was very useful for setting the NMR experiments at the pH values where the maximum formation of a given species was reached. When a histidine residue is present in a terminally blocked peptide, it normally acts as the primary binding site for the metal ions, through its imidazole nitrogen, while other ligands (usually water molecules or oxygen donors from the peptide, if present) are stepwisely substituted by deprotonated amide nitrogens from the backbone, giving rise to tetragonal 4N {N_*Im*⁡_, 3N^−^} species, as the pH is raised.

**Figure 2:**
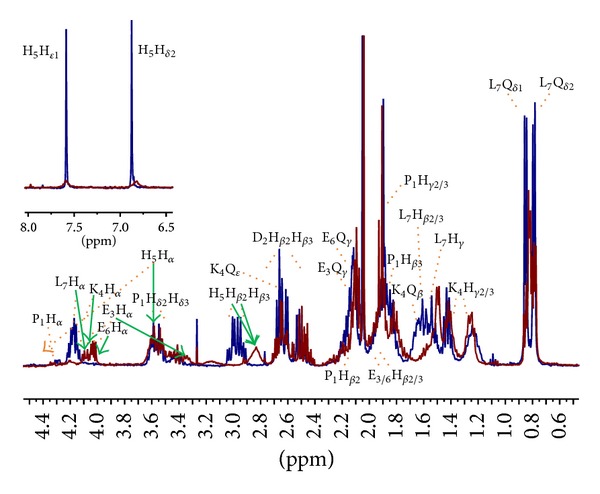
Superimposition of ^1^H NMR spectra for the PK9-H peptide, 2.5 mM, pH 11, and *T* 298 K, in the absence (blue) and in the presence (red) of 0.05 equivalents of Cu(II). New resonances due to Cu-binding have been indicated by green arrows.

With Cu(II) being a paramagnetic ion, 1D NMR spectra of Cu(II)/PK9-H system detected only a paramagnetic effect, giving a general broadening or disappearing for the resonances from the residues directly involved in the complex formation. In Figure 1, the titration of PK9-H peptide with increasing amounts of Cu(II) at pH 7.6 is shown. As inferred by potentiometric data [5], the species evidenced at this pH is of a {N_*Im*⁡_, 2N^−^, O} kind. In fact, in the aromatic region (Figure 1(a)), the disappearance of histidine protons suggests that coordination involves one of the imidazole nitrogens. Furthermore, the H*ε*1 signal appears to be more affected in comparison to H*δ*_2_, giving evidences of its closer distance to the paramagnetic centre, thus suggesting coordination to the metal ion through the N*δ*_1_ donor atom, as already found in other similar cases [30–32]. In the aliphatic region (Figure 1(b)), even if a general broadening is visible, the main residues affected by the metal ion are those related to His-5 and Lys-4, for which the aliphatic protons underwent the greatest effects. Clear evidence of the involvement of a carboxylic group in the coordination is also visible, and moreover, from the 2D ^1^H-^13^C HSQC NMR spectrum (Figure 2s), the complete vanishing of C*β*-H*β*2/3 from Asp-2 and the disappearance of C*γ*-Q*γ* signals from Glu-3 could confirm the {N_*Im*⁡_, 2N^−^, O} chromophore with the involvement of a carboxylic group in the coordination sphere, either from Asp-2 or Glu-3 residues, which could be in a dynamic equilibrium between them. The monodimensional spectra seem to indicate Glu3 as the residue most likely involved in the coordination.

**Figure 3:**
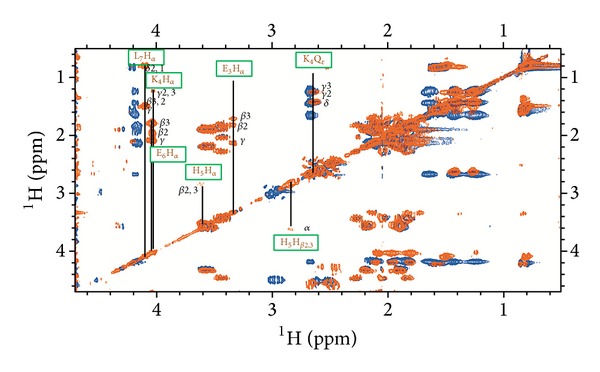
Selection of aliphatic region in the ^1^H-^1^H TOCSY NMR spectra for PK9-H peptide, 2.5 mM, pH 11, and *T* 298 K in the absence (blue) and in the presence (orange) of 0.05 equivalents of Cu(II). New resonances due to Cu-binding have been labelled.

**Figure 4:**
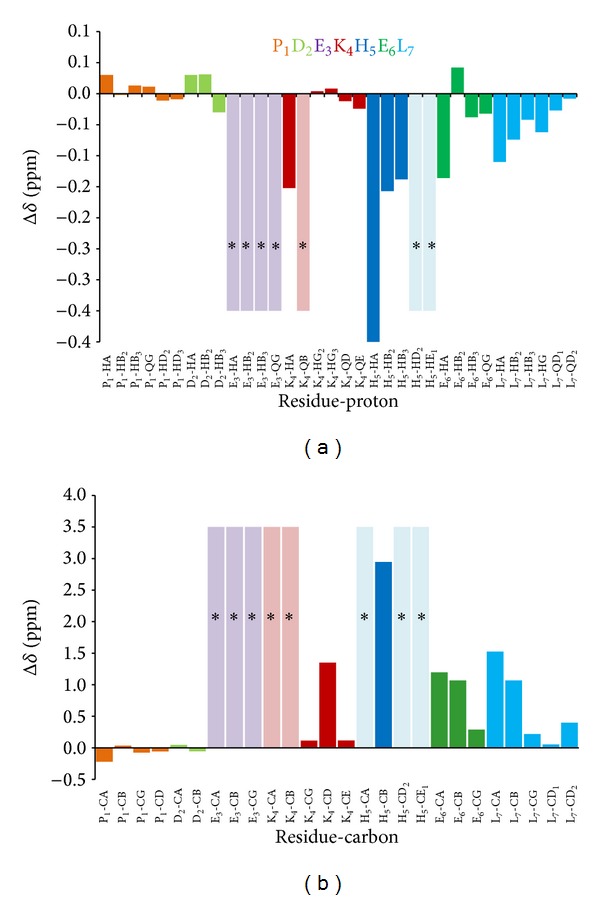
Plot of the observed ^1^H proton (a) and ^13^C carbon (b) chemical shift changes (Δ*δ* = *δ*_holo_ − *δ*_apo_) for PK9-H peptide following copper coordination at pH 11. Disappeared peaks have been labelled by asterisk*.

At basic pH, a further proton, belonging to H^N^ from Glu-3, is released, leading to the formation of a 4N {N_*Im*⁡_, 3N^−^} chromophore. In the NMR spectrum recorded at pH 11 (assignment for the free peptide at this pH is reported in Figure 3s), we simultaneously assist to the classical broadening and disappearing of some signals, due to the paramagnetic effects of the metal on the closest nuclei, plus a chemical shift variation involving the side chain protons and carbons of those residues taking part to metal coordination through their deprotonated amide nitrogens (Lys-4 and Glu-3). Both effects are clearly visible in the mono and bidimensional spectra in Figures 2 and 3, and in the plots of the observed proton and carbon chemical shift changes (Δ*δ* = *δ*_holo_ − *δ*_apo_) for PK9-H peptide/Cu(II) system at pH 11 (Figure 4 and Table  1s). 

In Figure 2, a superimposition of the free and bound peptide spectra is reported. A complete vanishing of His-5 imidazole signals in the aromatic region upon the addition of 0.05 equivalents of copper is seen, indicating that His-5 is the primary site for metal anchorage. At the same time, in the aliphatic region (Figures 2 and 3), His-5 side chain protons are still present, although slightly broadened and shifted upfield as a result of its H^N^ deprotonation (*Hα*Δ*δ* = −0.873 ppm, H*β*2/3 Δ*δ* = −0.157, and −0.138 ppm, resp.). On the other hand, all the resonances from Glu-3 together with Q*β* from Lys-4 disappeared.

**Figure 5:**
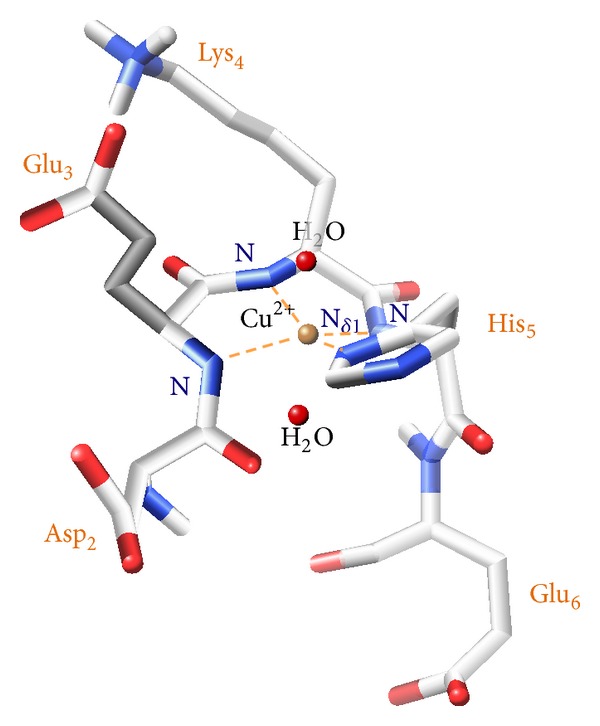
3D structural model for the Cu(II) ion complexed with PK9-H peptide in a distorted tetragonal geometry, with the involvement of the N*δ*_1_ imidazole nitrogen of the histidine residue, and three deprotonated amidic nitrogens from the peptide backbone (belonging to His-5, Lys-4, and Glu-3 residues), giving a {N_*Im*⁡_, 3N^−^} species.

The side chain of Glu-3 results repositioned over the coordination plane, while that of Lys-4 appears to lie out, far from the paramagnetic influence of the metal, as shown in the calculated model for this species (Figure 5). In fact, the latter residue shows a variable effect on its protons, with the shielding of H*α* and the disappearing of Q*β* signals that can be an indication of the closeness of this residue to the metal ion. Also a slight deshielding of H*γ*2/3 and again a shielding of Q*δ* and Q*ε* resonances can be observed. This finding might be due to the relative positioning of the side chain outside the coordination plane, in the opposite direction respect to the metal centre. The remaining residues, Pro-1, Asp-2, Glu-6, and Leu-7, although not directly participating in the complex formation, they are nevertheless influenced by its formation, as shown by a moderate to light shift of all their resonances, indicating that the conformational changes induced by metal coordination are able to affect also the farthest residues.

A final remark should be dedicated to Lys-4, since the differences in the chemical shifts of its aliphatic side chain protons, together with the disappearance, at pH 10, of C*γ*-H*γ*, while Q*δ* signals are still present, suggest the possible deprotonation of the terminal amino group of lysine residue though excluding its participation to metal coordination, as indicated also by the previous UV-Vis experiments [5]. No evidence of the involvement of Asp-2 carboxylic moiety into the coordination sphere has been found from NMR experiments at pH 10, given the poor effect of the metal on all its resonances which can be reflected by the slight chemical shift changes recorded for its protons.

### 3.2. Ni(II) Complex

We investigated the interaction of Ni(II) ion with PK9-H peptide in order to verify the peptide ability to bind this metal which, as a divalent cation, can be involved in cellular trafficking lead by Ypk9 protein and to gather more information about the PK9-H coordination behaviour.

In the NMR spectra recorded around neutral pH, Ni(II) results in a paramagnetic ion giving a general broadening or disappearing of the resonances from the residues directly involved in the complex formation and more specifically Asp-2, Glu-3/6 and His-5 (Figure 4s).

Usually, diamagnetic, low spin, square planar Ni(II) complexes are obtained at sufficiently high pH values, where the imidazole nitrogen of the histidine residue works as the anchoring site (in terminally blocked peptides) and the metal is able to deprotonate and bind three amide nitrogens from the backbone, thus completing its coordination sphere [29, 30, 32–35]. We recorded our spectra at pH 10.5, by slowly adding increasing amounts of Ni(II) ions, up to a PK9-H : Ni(II) 1 : 0.9 molar ratio. This titration allowed us to follow the changes undergone by the peptide upon nickel addition.

**Figure 6:**
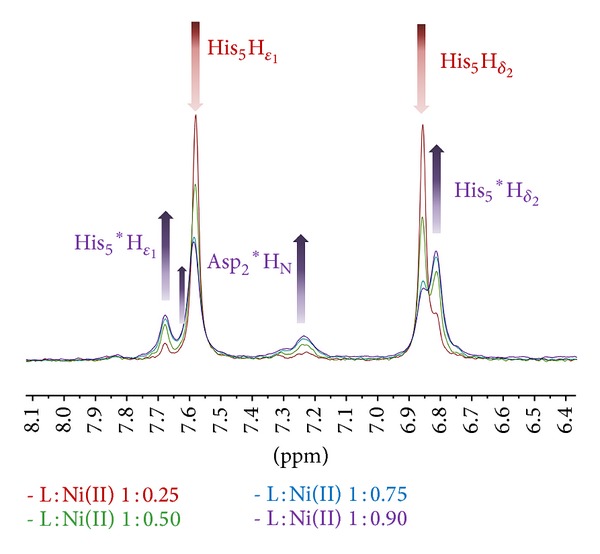
Superimposition of ^1^H NMR spectra in the aromatic regions for the PK9-H peptide, 2.5 mM, pH 10.5, and *T* 298 K, with increasing amounts of Ni(II) from 1 : 0.25 to 1 : 0.90 metal to ligand molar ratios. New resonances due to Ni-binding have been labelled by asterisk*.

Coordination to His-5 is immediately recognized by the gradual disappearing of the relative resonances in the free peptide (H*δ*_2_ and H*ε*_1_) and the concomitant appearing of a new set of signals for the bound state (Figure 6). In particular, it is possible to note that the appearance of two new signals corresponding to the H*ε*_1_ proton with the relative difference in Δ*δ* of less than 0.1 ppm. The main species, identified through the presence of more intense cross correlation signals in the TOCSY spectrum and ROEs peaks in the ROESY spectrum, is that with the chemical shift value for H*ε*_1_ at 7.602 ppm and slightly overlapping with the free signal of H*ε*_1_ which appears at 7.592 ppm.

The aromatic region reveals also the appearance of a new resonance belonging to Asp-2 H^N^ which becomes noticeable as the amount of nickel in solution increases. This is a first clue of the involvement of Asp-2 residue in the coordination sphere.

In the aliphatic region, on the other hand, we assist to some chemical shift changes which are more pronounced for the residues taking part to metal coordination, as expected for His-5, Lys-4, and Glu-3 side chains (Figure 5s). The general feature shown by their protons is a shielding effect for H*α*, due to the influence of an increased electron density upon amide deprotonation, a fate which is also shared by all the remaining protons in His-5 residue. Lys-4 resonances, instead, do not show a regular trend, with H*β*3 and H*γ*3 shifted upfield (probably blocked in a position which brings them above a shielding area of the complex, as shown also by the fact that their degeneracy is not retained after coordination), whereas the remaining part shifted down-field.

**Figure 7:**
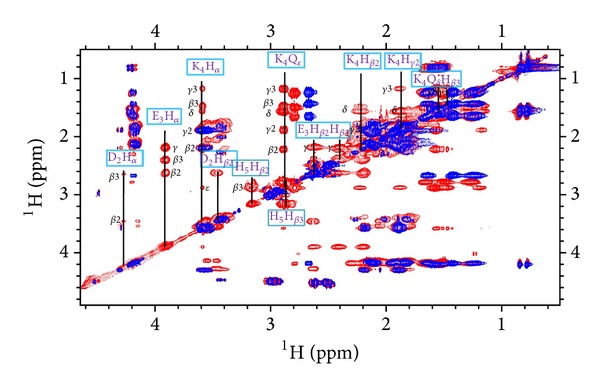
Selection of aliphatic region in the ^1^H-^1^H TOCSY NMR spectra for PK9-H peptide, 2.5 mM, pH 10.5, and *T* 298 K in the absence (blue) and in the presence (red) of 0.9 equivalents of Ni(II). New resonances due to Ni-binding have been labelled.

**Figure 8:**
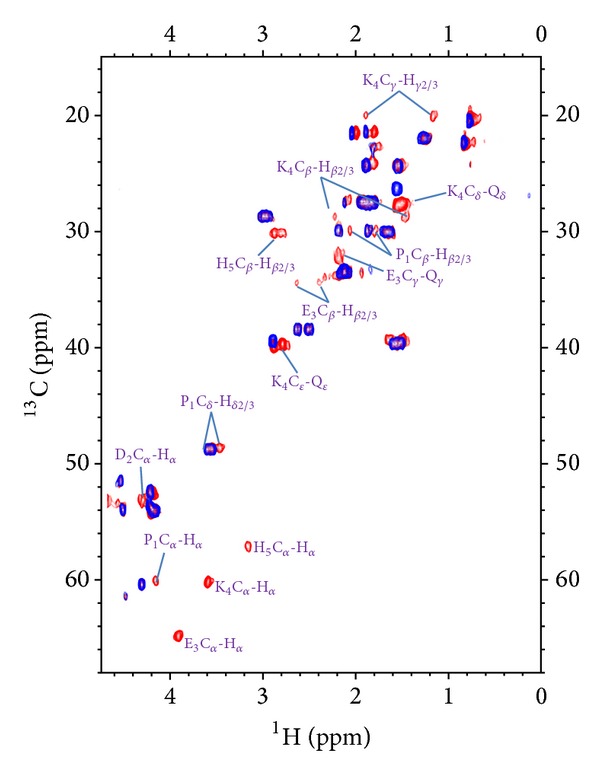
Selection of aliphatic regions in the ^13^C-^1^H HSQC NMR spectra for PK9-H peptide, 2.5 mM, pH 10.5, and *T* 298 K, in the absence (blue) and in the presence (red) of 0.9 equivalents of Ni(II). New resonances due to Ni-binding have been labelled.

**Figure 9:**
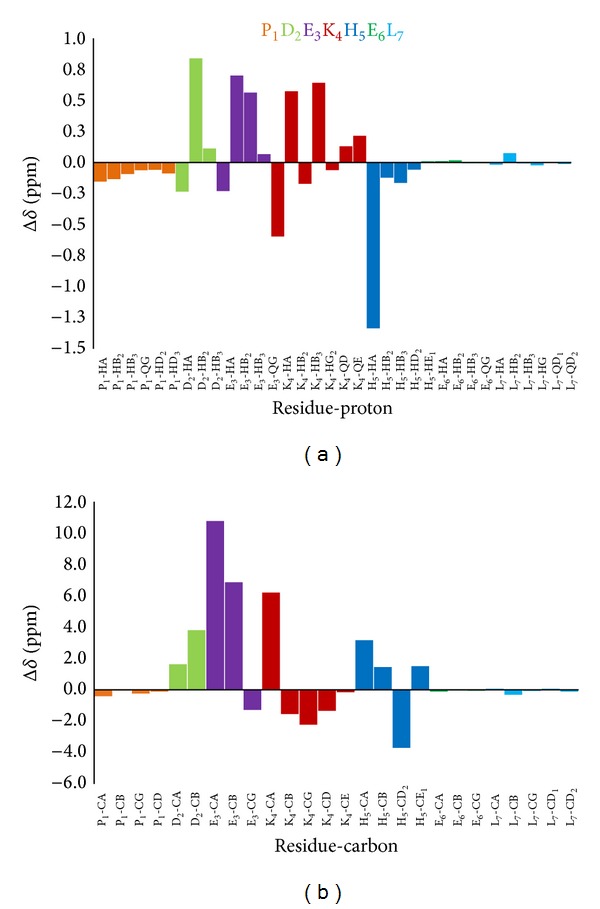
Plot of the observed ^1^H proton (a) and ^13^C carbon (b) chemical shift changes (Δ*δ* = *δ*_holo_ − *δ*_apo_) for PK9-H peptide following nickel coordination.

Glu-3 protons, on the other hand, are all deshielded with the order *β*2 > *β*3 > *γ*, probably due to a more rigid conformation adopted also by Glu-3 side chain within the complex. But the unexpected feature detected in the spectra is the marked chemical shift variation undergone by the Asp-2 protons, indicating that also this residue takes part in the complex formation. Precisely, the Δ*δ* trend indicates, at the same time, that the Asp-2 residue is involved in the coordination through its carboxylic group COO^−^ (Δ*δ* H*β*2/3 > H*α*) and that the large difference between the chemical shifts of two geminal beta proton (Δ*δ*H*β*2 ≫ H*β*3) is a symptom of a more rigid spatial conformation of the side chain. All these conclusions were clearly confirmed by the bidimensional spectra recorded and reported in Figures 7 and 8. In Figure 9, the plots of the observed proton and carbon chemical shift changes (Table  2s) for PK9-H peptide/Ni(II) system at pH 10.5 are shown.

ROESY experiments allowed us to collect a number of ROE contacts between the protons close in space, used to calculate a structural model for Ni(II)/PK9-H complex. In particular, some contacts were evidenced between H*β*2/3 and Q*γ* side chain protons from Glu-3 and H*ε*_1_ from His-5, showing that the Glu-3 residue is positioned towards the imidazole ring. Furthermore, also H*β* and H*α* from Asp-2 residue interact with the His-5 aromatic protons, suggesting the close proximity of Asp-2 residue to the coordination site.

**Figure 10:**
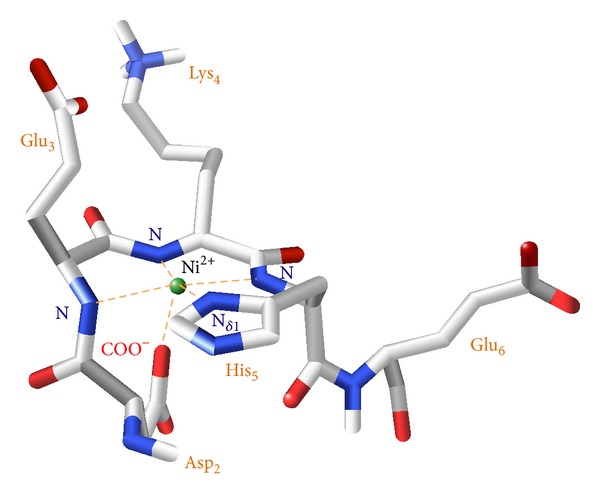
3D structural model, derived from NMR structural constraints (ROEs), for the Ni(II) ion complexed with PK9-H peptide in a pentacoordinated geometry, with the involvement of the N*δ*_1_ imidazole nitrogen of the histidine residue, three deprotonated amidic nitrogens from the peptide backbone (belonging to His-5, Lys-4, and Glu-3 residues), and the carboxylate moiety from aspartyl (Asp-2) residue in the apical position, giving a {N_*Im*⁡_, 3N^−^, O^−^} species.

It is thus clear that we are not simply facing a square planar species, as it could be expected, but as pointed out by the NMR data and the calculated model shown in Figure 10, we are able to state that also Asp-2 residue, through its carboxylate oxygen, is involved in the coordination to Ni(II) ion, thus forming a pentacoordinated {N_*Im*⁡_, 3N^−^, O^−^} species in a square pyramidal arrangement (*spy*). The energy levels distribution for the orbitals in a* spy* configuration should be qualitatively very similar to the square planar case (*d*_*yz*_ = *d*_*xz*_ < *d*_*z*2_ < *d*_*xy*_ ≪ *d*_*x*2−*y*2_), since the removal of only one ligand along the *z* axis of the octahedron introduces a perturbation qualitatively very similar to that caused by the removal, total or partial, of both ligands. A* spy* complex, with a fifth donor on the apical position (the COO^−^ group from Asp-2), can thus be compatible with the experimental data collected for this diamagnetic species.

This aspect clearly differentiates copper complexation, under the same pH conditions, from nickel, for which the presence in the coordination sphere of an additional donor was evidenced.

## 4. Conclusions

In this paper, we have completed the information about Cu(II) interaction with a possible divalent cations transporter, Ypk9 protein, through the study of one of its model peptides, P_1_D_2_E_3_K_4_H_5_E_6_L_7_ (PK9-H), a histidine-containing sequence. We have also studied the behaviour of Ni(II) ions, which are able to interact with the same sequence by forming a complex whose structural model has been build up on the basis of NMR data collected from the bidimensional experiments.

The results obtained in this study clearly show that each metal ion behaves according to its unique and peculiar features in binding to the same peptide; this fact is reflected in the formation of the different main species for the two ions. In fact, nickel is able to add to its coordination sphere an extra donor atom from the peptide, the oxygen from the carboxylate moiety of a nearby aspartate residue, yielding a square pyramidal coordination with a {N_*Im*⁡_, 2N^−^, O} instead of a tetragonal coordination with a {N_*Im*⁡_, 3N^−^} chromophore detected for copper. This occurrence must be held into consideration when dealing with metal-specificity of a given protein, as the one here examined.

Gathering the results we have collected for different divalent cations, such as Mn(II), Zn(II), Cu(II), and Ni(II) with a selected sequence of Ypk9 protein in which a possible effective site for metal binding, the histidine residue, is hosted, we are able to say that this member of the P_5_-type ATPases shows a remarkable attitude towards the studied cations, reinforcing the hypothesis of its role as a metal transporter agent.

## Supplementary Material

Supplementary Figures:
Figure 1s: Schematic stick representation of the PK9-H peptide with the proton labels.
Figure 2s: Selection of aliphatic regions in the 13C-1H HSQC NMR spectra for PK9-H peptide, 2.5 mM, pH 7.6, T 298 K, in the absence (red) and in the presence (blue) of 0.05 equivalents of Cu(II).
Figure 3s: 1D 1H NMR spectra of aliphatic and aromatic (inset) regions for PK9-H peptide, 2,5 mM, pH 11, T 298, with the relative chemical shift assignment.
Figure 4s: Stacked image of 1H NMR spectra for the aromatic a) and aliphatic b) regions for the PK9-H peptide, 2.5 mM, pH 6.9, T 298 K, in the absence (black) and in the presence of 0.05 equivalent of Ni(II). The disappearing resonances due to Ni-binding have been highlighted.
Figure 5s: Superimposition of 1H NMR spectra in the aliphatic regions for the PK9-H peptide, 2.5 mM, pH 10.5, T 298 K, with increasing amounts of Ni(II) from 1:0.0.25 to 1:0.90 metal to ligand molar ratios. New resonances due to Ni-binding have been labelled.
Supplementary Tables:
Table 1s: Chemical shifts assignment (ppm) for 1H and 13C nuclei of PK9-H peptide in the free and Cu2+ bound state (pH 11) and the relative chemical shifts differences (Δ*δ* = *δ*holo – *δ*apo).
Table 2s: Chemical shifts assignment (ppm)for 1H and 13C nuclei of PK9-H peptide in the free and Ni2+ bound state (pH 10.5) and the relative chemical shifts differences (Δ*δ* = *δ*holo – *δ*apo).
