# Supplementary material for: Interaction of Cu(II) and Ni(II) with Ypk9 Protein Fragment via NMR Studies
Source: ScientificWorldJournal. 2014 Mar 24;2014:656201. doi: 10.1155/2014/656201 (PMC3982466; doi:10.1155/2014/656201)
Supplement: Supplementary file 1 — Supplementary Figures: Figure 1s: Schematic stick representation of the PK9-H peptide with the proton labels. Figure 2s: Selection of aliphatic regions in the 13C-1H HSQC NMR spectra for PK9-H peptide, 2.5 mM, pH 7.6, T 298 K, in the absence (red) and in the presence (blue) of 0.05 equivalents of Cu(II). Figure 3s: 1D 1H NMR spectra of aliphatic and aromatic (inset) regions for PK9-H peptide, 2,5 mM, pH 11, T 298, with the relative chemical shift assignment. Figure 4s: Stacked image of 1H NMR spectra for the aromatic a) and aliphatic b) regions for the PK9-H peptide, 2.5 mM, pH 6.9, T 298 K, in the absence (black) and in the presence of 0.05 equivalent of Ni(II). The disappearing resonances due to Ni-binding have been highlighted. Figure 5s: Superimposition of 1H NMR spectra in the aliphatic regions for the PK9-H peptide, 2.5 mM, pH 10.5, T 298 K, with increasing amounts of Ni(II) from 1:0.0.25 to 1:0.90 metal to ligand molar ratios. New resonances due to Ni-binding have been labelled. Supplementary Tables: Table 1s: Chemical shifts assignment (ppm) for 1H and 13C nuclei of PK9-H peptide in the free and Cu2+ bound state (pH 11) and the relative chemical shifts differences (Δδ = δholo – δapo). Table 2s: Chemical shifts assignment (ppm)for 1H and 13C nuclei of PK9-H peptide in the free and Ni2+ bound state (pH 10.5) and the relative chemical shifts differences (Δδ = δholo – δapo). [file 656201.f1.zip › 656201.f1/Fig_1s_TSWJ_789312.pptx]

## Slide 1
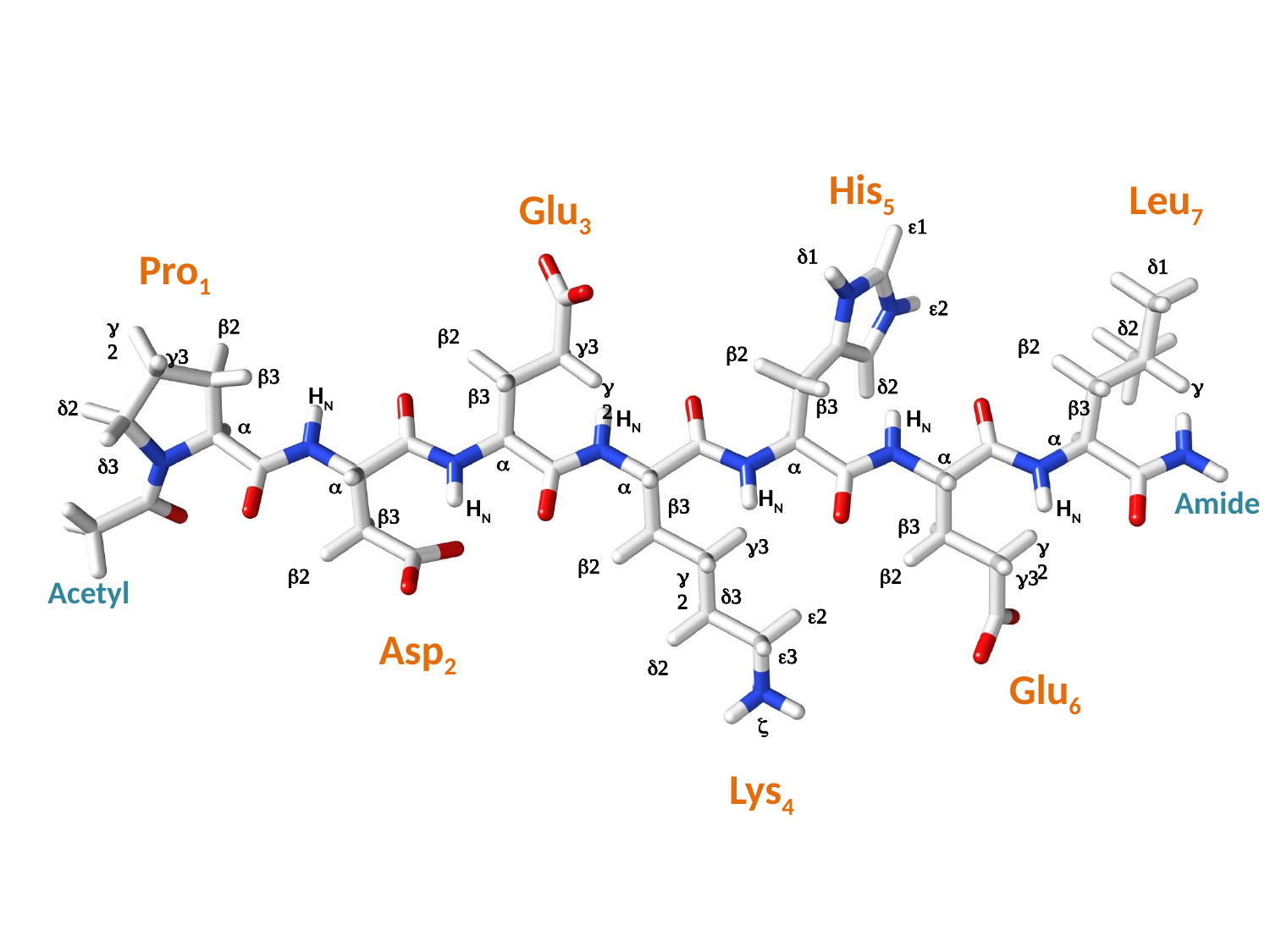

His5
Leu7
Glu3
e1
Pro1
d1
d1
e2
g2
b2
d2
b2
g3
b2
b2
g3
b3
g2
d2
g
HN
b3
b3
d2
b3
HN
HN
a
a
a
a
d3
a
a
a
HN
Amide
HN
b3
HN
b3
b3
g3
g2
b2
b2
g2
b2
g3
Acetyl
d3
e2
Asp2
e3
d2
Glu6
z
Lys4
