# Supplementary material for: Interaction of Cu(II) and Ni(II) with Ypk9 Protein Fragment via NMR Studies
Source: ScientificWorldJournal. 2014 Mar 24;2014:656201. doi: 10.1155/2014/656201 (PMC3982466; doi:10.1155/2014/656201)
Supplement: Supplementary file 1 — Supplementary Figures: Figure 1s: Schematic stick representation of the PK9-H peptide with the proton labels. Figure 2s: Selection of aliphatic regions in the 13C-1H HSQC NMR spectra for PK9-H peptide, 2.5 mM, pH 7.6, T 298 K, in the absence (red) and in the presence (blue) of 0.05 equivalents of Cu(II). Figure 3s: 1D 1H NMR spectra of aliphatic and aromatic (inset) regions for PK9-H peptide, 2,5 mM, pH 11, T 298, with the relative chemical shift assignment. Figure 4s: Stacked image of 1H NMR spectra for the aromatic a) and aliphatic b) regions for the PK9-H peptide, 2.5 mM, pH 6.9, T 298 K, in the absence (black) and in the presence of 0.05 equivalent of Ni(II). The disappearing resonances due to Ni-binding have been highlighted. Figure 5s: Superimposition of 1H NMR spectra in the aliphatic regions for the PK9-H peptide, 2.5 mM, pH 10.5, T 298 K, with increasing amounts of Ni(II) from 1:0.0.25 to 1:0.90 metal to ligand molar ratios. New resonances due to Ni-binding have been labelled. Supplementary Tables: Table 1s: Chemical shifts assignment (ppm) for 1H and 13C nuclei of PK9-H peptide in the free and Cu2+ bound state (pH 11) and the relative chemical shifts differences (Δδ = δholo – δapo). Table 2s: Chemical shifts assignment (ppm)for 1H and 13C nuclei of PK9-H peptide in the free and Ni2+ bound state (pH 10.5) and the relative chemical shifts differences (Δδ = δholo – δapo). [file 656201.f1.zip › 656201.f1/tables.doc]

**Supplementary information:**

**Tables**

**Table 1s.** Chemical shifts assignment (ppm) for ^1^H and ^13^C nuclei of PK9-H peptide in the free and Cu^2+^ bound state (pH 11) and the relative chemical shifts differences (Δδ = δ_holo_ – δ_apo_).

| **Residue-Proton** | **PK9-H free** | **PK9-H:Cu** | **Δδ ppm** |
| --- | --- | --- | --- |
| **P1 HA** | 4,300 | 4,330 | **0,030** |
| **P1 HB2** | 2,204 | 2,202 | **-0,002** |
| **P1 HB3** | 1,885 | 1,898 | **0,013** |
| **P1 QG** | 1,895 | 1,906 | **0,011** |
| **P1 HD2** | 3,602 | 3,591 | **-0,011** |
| **P1 HD3** | 3,545 | 3,536 | **-0,009** |
| **D2 HA** | 4,520 | 4,550 | **0,030** |
| **D2 HB2** | 2,625 | 2,656 | **0,031** |
| **D2 HB3** | 2,518 | 2,488 | **-0,030** |
| **E3 HA** | 4,132 | *disappeared* |  |
| **E3 HB2** | 1,921 | *disappeared* |  |
| **E3 HB3** | 1,843 | *disappeared* |  |
| **E3 QG** | 2,127 | *disappeared* |  |
| **K4 HA** | 4,178 | 4,026 | **-0,152** |
| **K4 QB** | 1,651 | *disappeared* |  |
| **K4 HG2** | 1,253 | 1,257 | **0,004** |
| **K4 HG3** | 1,239 | 1,247 | **0,008** |
| **K4 QD** | 1,436 | 1,424 | **-0,012** |
| **K4 QE** | 2,670 | 2,646 | **-0,024** |
| **H5 HA** | 4,495 | 3,622 | **-0,873** |
| **H5 HB2** | 3,004 | 2,847 | **-0,157** |
| **H5 HB3** | 2,972 | 2,834 | **-0,138** |
| **H5 HD2** | 6,880 | *disappeared* |  |
| **H5 HE1** | 7,592 | *disappeared* |  |
| **E6 HA** | 4,174 | 4,038 | **-0,136** |
| **E6 HB2** | 1,920 | 1,962 | **0,042** |
| **E6 HB3** | 1,841 | 1,803 | **-0,038** |
| **E6 QG** | 2,126 | 2,094 | **-0,032** |
| **L7 HA** | 4,209 | 4,099 | **-0,110** |
| **L7 HB2** | 1,575 | 1,501 | **-0,074** |
| **L7 HB3** | 1,528 | 1,486 | **-0,042** |
| **L7 HG** | 1,558 | 1,496 | **-0,062** |
| **L7 QD1** | 0,849 | 0,822 | **-0,027** |
| **L7 QD2** | 0,799 | 0,791 | **-0,008** |

| **Residue-Carbon** | **PK9-H free** | **PK9-H:Cu** | **Δδ ppm** |
| --- | --- | --- | --- |
| **P1 CA** | 60,424 | 60,203 | **-0,221** |
| **P1 CB** | 29,889 | 29,922 | **0,033** |
| **P1 CG** | 24,223 | 24,146 | **-0,077** |
| **P1 CD** | 48,702 | 48,644 | **-0,058** |
| **D2 CA** | 51,537 | 51,583 | **0,046** |
| **D2 CB** | 38,411 | 38,357 | **-0,054** |
| **E3 CA** | 54,068 | *disappeared* |  |
| **E3 CB** | 27,500 | *disappeared* |  |
| **E3 CG** | 33,479 | *disappeared* |  |
| **K4 CA** | 53,951 | *disappeared* |  |
| **K4 CB** | 30,259 | *disappeared* |  |
| **K4 CG** | 22,189 | 22,301 | **0,112** |
| **K4 CD** | 29,020 | 30,370 | **1,350** |
| **K4 CE** | 39,995 | 40,111 | **0,116** |
| **H5 CA** | 53,929 | *disappeared* |  |
| **H5 CB** | 28,654 | 31,599 | **2,945** |
| **H5 CD2** | 117,135 | *disappeared* |  |
| **H5 CE1** | 136,195 | *disappeared* |  |
| **E6 CA** | 53,984 | 55,180 | **1,196** |
| **E6 CB** | 27,510 | 28,578 | **1,068** |
| **E6 CG** | 33,507 | 33,796 | **0,289** |
| **L7 CA** | 52,486 | 54,010 | **1,524** |
| **L7 CB** | 39,638 | 40,706 | **1,068** |
| **L7 CG** | 24,324 | 24,541 | **0,217** |
| **L7 CD1** | 22,318 | 22,371 | **0,053** |
| **L7 CD2** | 20,454 | 20,851 | **0,397** |

**Table 2s.** Chemical shifts assignment (ppm)for ^1^H and ^13^C nuclei of PK9-H peptide in the free and Ni^2+^ bound state (pH 10.5) and the relative chemical shifts differences (Δδ = δ_holo_ – δ_apo_).

| **Residue-Proton** | **PK9-H free** | **PK9-H:Ni** | **Δδ ppm** |
| --- | --- | --- | --- |
| **P1-HA** | 4,298 | 4,145 | **-0,153** |
| **P1-HB2** | 2,204 | 2,071 | **-0,133** |
| **P1-HB3** | 1,885 | 1,793 | **-0,092** |
| **P1-QG** | 1,895 | 1,834 | **-0,061** |
| **P1-HD2** | 3,602 | 3,544 | **-0,058** |
| **P1-HD3** | 3,545 | 3,458 | **-0,087** |
| **D2-HA** | 4,514 | 4,281 | **-0,233** |
| **D2-HB2** | 2,626 | 3,465 | **0,839** |
| **D2-HB3** | 2,518 | 2,632 | **0,114** |
| **E3-HA** | 4,133 | 3,904 | **-0,229** |
| **E3-HB2** | 1,921 | 2,622 | **0,701** |
| **E3-HB3** | 1,844 | 2,408 | **0,564** |
| **E3-QG** | 2,124 | 2,192 | **0,068** |
| **K4-HA** | 4,178 | 3,584 | **-0,594** |
| **K4-HB2** | 1,651 | 2,225 | **0,574** |
| **K4-HB3** | 1,651 | 1,481 | **-0,170** |
| **K4-HG2** | 1,253 | 1,895 | **0,642** |
| **K4-HG3** | 1,239 | 1,179 | **-0,060** |
| **K4-QD** | 1,436 | 1,567 | **0,131** |
| **K4-QE** | 2,670 | 2,886 | **0,216** |
| **H5-HA** | 4,495 | 3,161 | **-1,334** |
| **H5-HB2** | 3,004 | 2,883 | **-0,121** |
| **H5-HB3** | 2,972 | 2,809 | **-0,163** |
| **H5-HD2** | 6,88 | 6,824 | **-0,056** |
| **H5-HE1** | 7,592 | 7,602 | **0,010** |
| **E6-HA** | 4,174 | 4,186 | **0,012** |
| **E6-HB2** | 1,920 | 1,938 | **0,018** |
| **E6-HB3** | 1,841 | 1,837 | **-0,004** |
| **E6-QG** | 2,126 | 2,122 | **-0,004** |
| **L7-HA** | 4,209 | 4,192 | **-0,017** |
| **L7-HB2** | 1,577 | 1,653 | **0,076** |
| **L7-HB3** | 1,529 | 1,523 | **-0,006** |
| **L7-HG** | 1,558 | 1,537 | **-0,021** |
| **L7-QD1** | 0,849 | 0,848 | **-0,001** |
| **L7-QD2** | 0,799 | 0,788 | **-0,011** |

| **Residue-Carbon** | **PK9-H free** | **PK9-H:Ni** | **Δδ ppm** |
| --- | --- | --- | --- |
| **P1-CA** | 60,423 | 60,027 | **-0,396** |
| **P1-CB** | 29,889 | 29,847 | **-0,042** |
| **P1-CG** | 24,223 | 23,995 | **-0,228** |
| **P1-CD** | 48,728 | 48,617 | **-0,111** |
| **D2-CA** | 51,537 | 53,171 | **1,634** |
| **D2-CB** | 38,411 | 42,219 | **3,808** |
| **E3-CA** | 54,068 | 64,835 | **10,767** |
| **E3-CB** | 27,500 | 34,368 | **6,868** |
| **E3-CG** | 33,479 | 32,208 | **-1,271** |
| **K4-CA** | 53,951 | 60,156 | **6,205** |
| **K4-CB** | 30,259 | 28,72 | **-1,539** |
| **K4-CG** | 22,189 | 19,972 | **-2,217** |
| **K4-CD** | 29,020 | 27,692 | **-1,328** |
| **K4-CE** | 39,995 | 39,853 | **-0,142** |
| **H5-CA** | 53,935 | 57,101 | **3,166** |
| **H5-CB** | 28,654 | 30,106 | **1,452** |
| **H5-CD2** | 117,135 | 113,439 | **-3,696** |
| **H5-CE1** | 136,195 | 137,704 | **1,509** |
| **E6-CA** | 53,984 | 53,877 | **-0,107** |
| **E6-CB** | 27,509 | 27,479 | **-0,030** |
| **E6-CG** | 33,507 | 33,453 | **-0,054** |
| **L7-CA** | 52,486 | 52,557 | **0,071** |
| **L7-CB** | 39,643 | 39,339 | **-0,304** |
| **L7-CG** | 24,324 | 24,266 | **-0,058** |
| **L7-CD1** | 22,318 | 22,385 | **0,067** |
| **L7-CD2** | 20,454 | 20,344 | **-0,110** |
